# Antibiotic Mixtures in Noninstrumental Endodontic Treatment of Primary Teeth with Necrotic Pulps: A Systematic Review

**DOI:** 10.1155/2021/5518599

**Published:** 2021-05-27

**Authors:** Farah Chouchene, Fatma Masmoudi, Ahlem Baaziz, Fethi Maatouk, Hichem Ghedira

**Affiliations:** ^1^Pediatric and Preventive Dentistry Department, Faculty of Dental Medicine of Monastir, Monastir, Tunisia; ^2^Laboratory of Biological Clinical and Dento-Facial Approach (ABCDF Laboratory LR12ES10), University of Monastir, Monastir, Tunisia

## Abstract

**Objective:**

To compare the effectiveness of topical antibiotic mixtures used in noninstrumental endodontic treatment (NIET) of primary teeth.

**Methods:**

Electronic databases including MEDLINE, the Cochrane Library, and Scopus database were searched. Randomized clinical trials evaluating the clinical and radiological outcomes of topical antibiotics used in NIET were selected. The revised Cochrane risk-of-bias tool (RoB 2.0) was used to assess the quality of the methodology of the included articles.

**Results:**

Five articles comparing the outcomes of four different drugs combination were included. Three studies conducted to evaluate the success rate of two combinations of antibacterial drugs consisting of ciprofloxacin-minocycline-metronidazole (3 Mix) in one group and ciprofloxacin-minocycline-ornidazole in the other group showed no statistically significant difference between both groups (*P* > 0.05). The ciprofloxacin-minocycline-ornidazole group showed better results compared with the 3 Mix group. One study conducted to compare the effectiveness of 3 Mix with ciprofloxacin-tinidazole-minocycline reported no significant difference between both groups, and one study that compared 3 Mix and ciprofloxacin-metronidazole-clindamycin mixture concluded that the overall success rates of both groups were 80.96% and 76.20%, respectively, with no statistically significant difference.

**Conclusion:**

Based on the overall success rates, the ciprofloxacin-minocycline-ornidazole mixture was considered more effective than the 3 Mix which was more effective than the ciprofloxacin-tinidazole-minocycline and the ciprofloxacin-metronidazole-clindamycin groups. *Clinical Relevance*. Different antibiotic combinations, showing good clinical and radiographic success in treating necrotic primary teeth, can be used effectively in NIET and this technique can be considered effective for teeth with advanced root resorption and when conventional endodontic treatment is contraindicated.

## 1. Introduction

Due to its complex root canal system, root resorption, difficulty in mechanical debridement, and polymicrobial nature of the infection, the successful management of chronically infected primary teeth is a challenge [1], but if the pathological process can be achieved, the preservation of the primary tooth is the best space maintenance for its successor. The liability to reinfection and the difficulties of complete root canal disinfection can make the prognosis less advantageous in chronically infected teeth [2].

In an infected root canal, the microbial flora is generally composed of both aerobic and anaerobic bacteria, but it is mainly anaerobic bacteria that most colonize the infected canals [3]. Therefore, during root canal debridement, practitioners must clean and disinfect the canals to eradicate these bacteria [2]. During both chemical and mechanical disinfection, several drugs can be used. It has been reported by Sato et al. [2] that disinfection of root canals with various antiseptics and/or antibiotics provides additional disinfection of about 20–40% of conventional root canal debridement. Several drugs such as antibiotics and antiseptics can be used for debridement, and the selection of those drugs with antibacterial action should be updated to help practitioners choose the best root canal sterilization drugs. Thus, the antibiotics may be useful in endodontic treatment and root canal sterilization of infected primary teeth and could eliminate all the possible bacteria from lesions [2].

The Cariology Research Unit of the Niigata University, School of Dentistry in Japan, has developed the concept of “noninstrumentation endodontic treatment” (NIET) also called “lesion sterilization and tissue repair” (LSTR) in which “no mechanical instrumentation was used” involves topical application of a mixture of three antibiotics: metronidazole, minocycline, and ciprofloxacin (3 Mix) mixed with propylene glycol and polyethylene glycol as a carrier, the so-called 3 Mix-MP [4, 5]. This mixture could sterilize infected necrotic pulp and root dentin in primary teeth. However, the presence of minocycline can lead to discoloration; thus, it was necessary to investigate the efficacy of a substitute mixture that is minocycline-free but may be able to produce the same outcomes in comparison with the original mixture [6].

Many mixtures have been proposed over the years and the present review aimed to compare the effectiveness of several antibiotic mixtures used in NIET of primary teeth with necrotic pulp.

## 2. Methods

### 2.1. Protocol and Registration

The present review was reported according to the principles of the Preferred Reporting Items for Systematic Reviews and Meta-Analysis (PRISMA) statement [7] and the Cochrane Handbook [8]. The protocol was registered at the International Prospective Register of Systematic Reviews (PROSPERO) under protocol ID CRD42020205621.

### 2.2. Review Question

The focused question was based on the Participants, Interventions, Control, Outcomes and, Study design (PICOS) principles: “In NIET of primary teeth with necrotic pulps, which antibiotic mixture was the best choice?” The detailed PICOS principles were as follows: (1) Participants: children having primary teeth with necrotic pulps. (2) Interventions: NIET with topical application of modified antibiotic mixtures (modified 3 Mix paste). (3) Control: NIET using an antibiotic mixture consisting of ciprofloxacin-minocycline-metronidazole (original 3 Mix paste). (4) Outcome: clinical and radiographic success rates. (5) Study design: randomized control trials.

### 2.3. Eligibility Criteria

Only studies reporting both clinical and radiographical outcomes of topical antibiotics used in noninstrumentation endodontic treatment (NIET) for primary teeth were considered eligible.

The studies were selected based on the following inclusion criteria:

- Randomized clinical trials (RCTs) reporting the clinical and radiographical outcomes of two mixtures of three topical antibiotics, the antibiotic mixture consisting of ciprofloxacin-minocycline-metronidazole (original 3 Mix paste) to a modified antibiotic mixture, used in NIET conducted in primary teeth with a necrotic pulp of healthy children.
- Studies reporting a clear evaluation of treatment outcomes based on both clinical and radiographic evaluation with at least 12-month follow-up period.

The studies were excluded based on the following exclusion criteria:

- Case reports, reviews, cross‐sectional, retrospective, prospective, nonrandomized, in vitro, and animal studies.
- Studies investigating pulpotomy and pulpectomy in primary teeth and permanent teeth and studies including primary teeth sustaining traumatic i/njuries and primary teeth without succedaneums were excluded from the present review.

### 2.4. Search Strategy

Search strategies were designed to identify studies discussing both the clinical and the radiographic outcomes of the application of topical antibiotic paste in primary teeth. Three electronic databases, MEDLINE (via PubMed), The Cochrane Library (CENTRAL), and Scopus, were searched for articles published in English published between 2000 and 2020. An initial search was performed in October 2019 and a subsequent search was achieved in April 2020.

The following search terms and combinations of Medical Subject Heading (MeSH) terms and keywords/text words were used and adapted for each database: (Root Canal Preparation OR Root Canal Therapy OR Root Canal Treatment OR LSTR OR NIET OR Non-instrumentation Endodontic Treatment) AND (Metronidazole OR Ciprofloxacin OR Minocycline OR Tinidazole OR Tetracycline OR Anti-Bacterial Agents OR Agents Anti-Bacterial OR Agents Antimycobacterial OR Antibiotic OR Antibiotic Paste OR Antibacterial Drugs) AND (Tooth, Deciduous). The set of keywords used during the search is summarized in Table 1.

To be certain to carry out a complete search on the concerned subject, a manual research in addition to the first electronic search was performed. The research was then supplemented by tracking citations of relevant studies via Google Scholar. To avoid any risk of bias, a gray literature search via opengrey.eu was also carried out by the two authors (FC and FM) to identify any additional unpublished studies.

### 2.5. Studies Selection

All the records selected from the different databases were managed by the EndNote X9 software (Clarivate, London, UK). Two authors (FC and FM) independently reviewed the titles of all studies. After title selection, the two authors reviewed all the abstracts to identify potentially eligible studies. The studies were excluded when no clinical and/or radiographic outcomes of the application of topical antibiotic paste in NIET were discussed. The selected studies were downloaded as full-text papers and then screened by the two authors. All eligible studies' references were also examined. A senior author (AB) resolved disagreements by discussion.

### 2.6. Data Extraction

Authors (FC and FM) collected data from the eligible studies separately using a standard pilot extraction sheet. The following items were summarized: variables including publication details (first author name, year of publication, and country), study methodology (study design, sample size, age of children, number of teeth, tooth type, and type of antibiotics mixture), follow‐up period, clinical and radiographical outcomes, and statistical significance of success rates. All the studies were subject to qualitative analyses.

In the present review, the success of treatment depended on clinical and radiographic outcomes measured according to established criteria. There were no restrictions on the maximum follow-up period or sample size. The clinical success was considered when there is no pain, no swelling, no abscess, no pain on percussion, and/or decrease in mobility. The radiographical success was considered as an absence or decrease of radiolucency when comparing x‐rays taken postoperatively with preoperative imaging. No change in radiolucency was also considered as an indicator of radiographical success. As based on an adaptation of Strindberg's criteria [9] and following a core set of component outcomes to define failure of a pulp treatment proposed by Smaïl-Faugeron et al. [10, 11], the treatment was considered a failure if one of the following symptoms was reported.

To determine heterogeneity, the following variables were checked: NIET procedure, antibiotics mixture, restoration materials, and outcomes variables.

### 2.7. Quality Assessment

Two authors (FC and FM) independently assessed the quality of the methodology and the results outcomes of the included studies using the revised Cochrane risk-of-bias tool for randomized trials (RoB 2.0) [12]. To assess each included trial for risk of bias, the following five domains were rated: (D1) randomization process; (D2) deviations from intended interventions; (D3) missing outcome data; (D4) measurement of the outcomes; and (D5) selection of the reported results. The RoB 2.0 was conceived hierarchically and authors must answer the signaling questions that provide the basis for domain-level judgements about the risk of bias and evaluate the overall bias of each included study according to the algorithm explained by RoB 2.0 guidance.

## 3. Results

### 3.1. Study Selection

**Figure 1:**
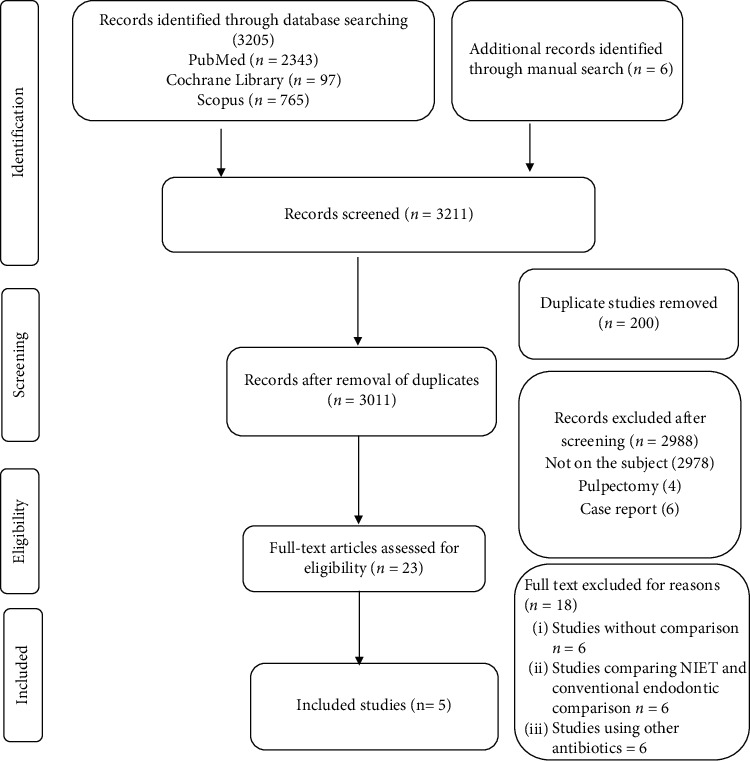
Prisma flow diagram.

The searches yielded 3,211 potentially related titles (Figure 1). A total of 200 duplicate references were removed. After title and abstract screening, 2,988 studies were excluded. The remaining 23 articles were selected for a full-text review. After the full-text analysis, 18 studies were eliminated based on the exclusion criteria, and five articles were included for assessment.

### 3.2. Study Characteristics

The five RCTs included in the present review were published between 2011 and 2017; four were conducted in India [13–16], and one was conducted in Syria [17].

A total of 232 primary teeth (with clinical characteristics showing one or more signs and symptoms indicating pulpectomy) of 151 cooperative children aged between 4 and 10 years were pulpectomized and had follow-up periods ranging from one to 24 months.

The included studies had similar study approaches. Variations were present essentially in the used type of antibiotic mixture. Three studies [13, 14, 16] were conducted to evaluate the clinical and radiographic success of NIET of infected primary teeth using two combinations of antibacterial drugs consisting of ciprofloxacin-minocycline-metronidazole (3 Mix) in one group and ciprofloxacin-minocycline-ornidazole in the other group. One study was conducted to compare the clinical and radiographic effectiveness of 3 Mix with ciprofloxacin-tinidazole-minocycline [15], and one study compared 3 Mix and ciprofloxacin-metronidazole-clindamycin mixture [17]. The used drug ratio was 1 : 3 : 3 in four studies [13–16] and 1 : 1 : 1 in one study [17]. Carriers, macrogol or macrogol and propylene glycol, were added to the different antibiotics powder mixtures until a consistent paste was procured. All the clinical procedures were performed under rubber dam isolation. In each tooth, a cavity was prepared depending on the extent of the lesion and caries were removed with no overhanging tooth structure left, to provide good access to the coronal pulp. Only the pulp chamber was removed, and no instrumentation of the canal was performed on the included teeth. Irrigation with saline was performed in four studies [13–16] and with 35% phosphoric acid (60 s) and sterile water in one study [17]. Selected teeth were sealed with glass ionomer cement and then restored with stainless steel crowns in one visit in three studies [14, 15, 17], filled with a temporary dressing (zinc oxide eugenol) and then restored with stainless steel crowns after 30 days in one study [13], and filled with a temporary dressing and then with permanent restoration after 15 days and restored with stainless steel crowns after 30 days in one study [16]. Clinical follow-ups were performed at 1, 3, 6, and 12 months in one study [17], at 6, 12, and 24 months in one study [15], and at 3–6 and 12 months in three studies [13, 14, 16].

### 3.3. Risk of Bias

**Figure 2:**
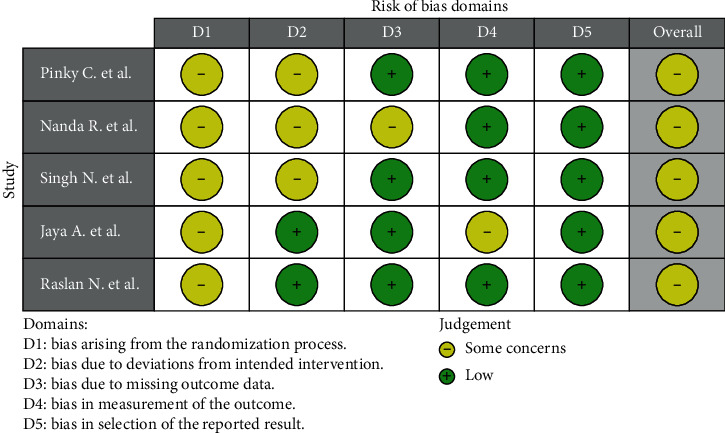
Risk-of bias summary (green indicates low risk of bias and yellow indicates some concerns of bias).

The risk-of-bias assessment summarized in Figure 2 was generated by the robvis (visualization tool) which is a web application designed for visualizing risk-of-bias assessments [18]. All the included studies had some concerns of overall bias and the most perplexing domain was the randomization process. All the included studies showed some concerns of bias in this domain because of the lack of randomization implementation. The methods of randomization were not explained in all the included studies. Three studies presented some concerns because of adhering to interventions. Evaluation of clinical and radiographic outcomes involved some examiner judgement, and minimizing the potential bias blinding was necessary. However only one included study specified that the examiners were blinded. Only the risk of bias in the selection of the reported results was considered low. Given the high data heterogeneity and the low number of the included studies, the fulfillment of quantitative analyses was not considered.

### 3.4. Main Outcomes

Five published articles comparing the potential outcomes of metronidazole, minocycline, and ciprofloxacin (3 Mix) with three different drugs' combinations were included in the present review (Tables 2 and 3):

  Comparison 1: 3 Mix versus ciprofloxacin-minocycline-ornidazole mixture.
  At 3-, 6-, and, 12-month follow-up, according to Pinky et al. [13], Nanada et al. [17], and Singh et al. [16], no statistically significant difference was reported (*P* > 0.05) when comparing the clinical and radiographical success rates between ciprofloxacin-minocycline-metronidazole mixture (3 Mix) and ciprofloxacin-minocycline-ornidazole mixture; both groups showed 100% clinical success, whereas the radiographic success rate of the ciprofloxacin-minocycline-ornidazole mixture was higher than 3 Mix.
  Comparison 2: 3 Mix versus ciprofloxacin-tinidazole-minocycline mixture.
  According to Jaya et al. [15], after 24-month follow-up, no statistically significant difference in the clinical and radiographical success rates between 3 Mix and the mixture of ciprofloxacin-tinidazole-minocycline was reported, but a higher radiographic success rate was observed with the 3 Mix.
  Comparison 3: 3 Mix versus ciprofloxacin-metronidazole-clindamycin mixture.

Raslan et al. [17] founded that the overall success rates of the 3 Mix group and the ciprofloxacin-metronidazole-clindamycin group were 80.96% and 76.20%, respectively, with no statically significant difference (*P* > 0.05).

Regarding the appearance of radiolucency and radiographic success rate, no statistically significant differences were noticed between the two groups after 6 and 12 months of treatment (*P* > 0.05).

## 4. Discussion

Due to the atypical primary tooth morphology, difficulties due to root canal fillings materials, and especially the complexity of root canals, endodontic treatment in primary teeth has been always considered a challenge for clinicians. Therefore, it is sometimes necessary to use an antibacterial drug capable of penetrating the tissues to be able to control and reduce the infections in nonvital infected primary teeth [2, 19]. Because the bacterial composition of the infected root canal was considered complex and the infection was designed to be a polymicrobial infection, a single antibacterial drug may not be effective; for that reason, several combinations of medicaments were tried over the years [13].

A combination of three antibiotics, metronidazole, ciprofloxacin, and minocycline (3 Mix), was shown to be promising. Although none of these drugs resulted in complete elimination of the bacterial composition of the infected root canal, several studies have shown that in combination these three drugs were able to consistently sterilize all the infected canals [13, 20].

Metronidazole has been the best drug in root canal disinfection since the root canal wall often has infected dentin colonized primarily by anaerobic bacteria. But metronidazole alone, even at high concentrations, cannot eradicate all the intracanal microbial flora, which has forced researchers to combine it with other drugs to increase its effectiveness and properly eliminate all bacteria. Thus, ciprofloxacin and minocycline have been added to metronidazole to obtain better results and eliminate all the microorganisms from the infected canals [2]. Furthermore, this antibiotics combination has been successful not only in primary teeth root canals sterilization but also in regenerative endodontic treatment and permanent tooth disinfection [21, 22].

Sato et al. [2] reported that the 3 Mix paste produced effective destruction of all aerobic and anaerobic endodontic pathogens. An in vitro study, conducted by Adl et al. [23], comparing the antibacterial effects of the 3 Mix paste and the calcium hydroxide paste against *Enterococcus faecalis (E. faecalis)*, showed that the 3 Mix paste was very effective and can be considered as more powerful root canal medicament compared to calcium hydroxide pastes.

These results suggest also that the 3 Mix paste with either 2% chlorhexidine or normal saline would be the most effective drug against *E. faecalis* and among its three components, the minocycline showed the greatest antibacterial effect.

The combination of the ciprofloxacin-ornidazole-minocycline showed 100% success rate which may be attributed to the use of ornidazole instead of metronidazole. In fact, ornidazole showed a longer duration of action with better efficacity and slower metabolism compared with metronidazole [13, 14, 16].

Comparing the relative efficacity of metronidazole and tinidazole in combination with ciprofloxacin and minocycline, Jaya et al. [15] reported that tinidazole can present several advantages over metronidazole including greater potency against both sensitive and resistant strains of obligate anaerobes with more prolonged action duration and improved patient tolerability.

Sensitization and or hypersensitivity reaction is one of the risks of the application of antibiotics; however, in all the included studies, none of the cases showed any evidence of such possible reactions to all the used antibiotics combination.

The 3 Mix can properly sterilizes carious lesions, necrotic pulps, and infected root dentin; however, the presence of minocycline can cause discoloration [17]. It was therefore important to investigate the efficacy of a substitutional mixture minocycline-free inducing the same outcomes. For that reason, some authors made changes in the 3 Mix paste, replacing the minocycline with clindamycin which can be considered as one of two options for patients with allergic reactions to penicillin and cephalosporin antibiotics in pediatric dentistry, and since it is effective for infections caused especially by Gram-positive aerobic bacteria and Gram-positive or Gram-negative anaerobic bacteria, clindamycin can be considered as a very interesting substitute [21, 24, 25]. According to Raslan et al. [17], both the 3 Mix paste and the ciprofloxacin-metronidazole-clindamycin combination can be effective in endodontic treatment. However, the 3 Mix paste showed a higher overall success rate.

The propylene glycol with and without macrogol was used for delivery of triple antibiotics paste in all the included studies in this review. This vehicle can act as a solvent enhancing better diffusion of the drugs deep into the dentinal tubules, thus enhancing the antimicrobial action which explains their use in the majority of the selected studies [26].

According to these findings, the 3 Mix group, the ciprofloxacin-minocycline-ornidazole group, the ciprofloxacin-tinidazole-minocycline group, and the ciprofloxacin-metronidazole-clindamycin group showed excellent clinical success, whereas the radiographic success rate of the ciprofloxacin-minocycline-ornidazole mixture was higher than that of 3 Mix group, which was higher than that of the ciprofloxacin-tinidazole-minocycline and the ciprofloxacin-metronidazole-clindamycin groups.

As it is known, restorative failure can influence the performance of endodontic treatment and the tooth must be restored at the end of any endodontic treatment to prevent microleakage at the restoration-tooth interface [27].

In the present review, all the included teeth were restored with glass ionomer cement and then with stainless steel crowns; however, previous studies evolving primary teeth have confirmed that not only the type of material but also the time elapsed between the temporary and the final restoration can be considered as parameters closely influencing the outcomes of endodontic treatment.

The five included studies used stainless steel crowns as a final restorative material, while the time between the treatment and the final restoration ranged from the same appointment after 15 days and even 30 days later.

In all the selected article in the present review, the inclusion criteria were teeth showing pain or tenderness to percussion, abscess, fistula, or clinical mobility that is incongruent with the physiological root resorption, evidence of periapical/bifurcation radiolucency, pathological external root resorption, or excessive bone resorption on radiographs, which indicates that NIET with topical application of a mixture of three antibiotics was effective for teeth with poor prognosis and when conventional endodontic treatment was contraindicated [28–30].

To better standardize the studies comparison in the present review, papers reporting NIET procedures different from the standard described method in the literature were excluded (the nonuse of rubber dam, the use of mechanical instrumentation, the use of a paste of a single antibiotic paste, and several agents used for hemostasis that could act as bias on the clinical outcomes).

The studies included in the present review evaluated the effectiveness of different combination of antibiotic pastes which were applied with almost the same procedure; however, these pastes were mixed by different drugs produced by different laboratories and may have a slightly different composition.

Almost the same coronal restorative materials were used to restore the treated teeth (glass ionomer cement; stainless steel crowns). However, to avoid any additional risk of bias, studies describing temporary materials used for tooth restoration were excluded.

All the included studies assessed both clinical and radiographic variables and the success criteria chosen by the authors were similar but not the same.

It was not possible to make a descriptive comparison between included studies, because although all the included studies assessed both clinical and radiographic variables, the success criteria chosen by the authors were similar but not the same.

## 5. Limitations

Some included studies did not fully describe the employed methods for sample-size calculation, randomization, blinding, and patient dropout control.

The small number of the included studies and the small sample size of these studies can be considered as an important limitation of this review. In the present review, most comparisons occurred based on single studies and this could influence the accuracy of the conclusion. The difference observed across the included studies may have stemmed from systematic differences within the studies analysed.

The used mixture of three broad-spectrum antibiotics, namely, metronidazole, ciprofloxacin, and minocycline (3 Mix), has shown good clinical and radiographic success in the treatment of primary teeth with necrotic pulp but for patients with allergic reactions, the modification of 3 Mix by using clindamycin in place of minocycline and ornidazole or tinidazole in place of metronidazole has also shown a good clinical result.

However, based on the present limited evidence, it was difficult to draw any conclusion as to the benefits of an antibiotic mixture over another. But whatever the used mixture of antibiotics, the results of the studies included in the present review have shown that this technique can be considered effective for teeth with advanced root resorption and when conventional endodontic treatment is contraindicated. Future clinical trials with longer follow-up periods and larger sample sizes are needed before a reliable conclusion can be drawn as to the best antibiotics mixture in primary teeth pulpectomy.

## Figures and Tables

**Table 1:** Keywords used to develop the search strategies.

Database	Keywords	*N*
PubMed	((“Dental pulp Necrosis”[Mesh]) AND “anti-bacterial Agents”[Mesh]) AND “tooth, Deciduous”[Mesh] ((“tooth, Deciduous”[Mesh]) AND “root canal Therapy”[Mesh]) AND “anti-bacterial Agents”[Mesh] ((“anti-bacterial Agents”[Mesh]) AND “tooth, Deciduous”[Mesh] ((“anti-bacterial Agents”[Mesh]) AND “root canal Preparation”[Mesh]) AND “tooth, Deciduous”[Mesh] ((“root canal Therapy”[Mesh]) OR “root canal preparation”[Mesh]) AND “anti-bacterial Agents”[Mesh] AND “tooth, Deciduous”[Mesh] ((“Metronidazole”[Mesh]) OR “Ciprofloxacin”[Mesh]) AND “Minocycline”[Mesh]) AND “Tinidazole”[Mesh]) AND “Tetracycline”[Mesh]) AND “Ornidazole”[Mesh]) AND “Clindamycin”[Mesh]) OR “anti-bacterial Agents”[Mesh]) AND (“root canal Preparation”[Mesh] OR “root canal Therapy”[Mesh])) AND “tooth, Deciduous”[Mesh]	2343

Cochrane Library	“#1 dental pulp necrosis”	97
	“#2 anti-bacterial agents”	
	“#3 root canal therapy”	
	“#4 root canal preparation”	
	“#5 deciduous tooth”	
	“#6-#1 AND #2 AND #3”	
	“#7-#6 AND #3 AND #2”	
	“#8-#5 AND #2 AND #6”	
	“#9-#2 AND #4 AND #6”	
	“#10-#3 OR #3 AND #2 AND #6”	
	“#11 metronidazole”	
	“#12 ciprofloxacin”	
	“#13 minocycline”	
	“#14 tinidazole”	
	“#15 tetracycline”	
	“#16 ornidazole”	
	“#17 clindamycin”	
	“#18 agent antimycobacterial”	
	“#19 antibiotic”	
	“#21 antibiotic paste”	
	“#22 non-instrumentation endodontic”	
	“#23 lstr”	
	“#24 root canal treatment”	
	“#25-#12 OR #12 OR #13 OR #14 OR #15 OR #16 OR #17 OR #18 OR #19 OR #20 OR #21 AND #5 AND #6”	
	“#26-#5 OR #3 OR #4 OR #22 OR #23 OR #24 AND #6”	

Scopus	TITLE-ABS-KEY (“Dental pulp Necrosis” AND “Anti-Bacterial Agents” AND “Tooth, Deciduous”)	765
	TITLE-ABS-KEY (“tooth, deciduous” AND “root canal therapy” AND “anti-bacterial agents”)	
	TITLE-ABS-KEY (” anti-bacterial agents” AND “tooth, deciduous”)	
	TITLE-ABS-KEY (“anti-bacterial agents” AND “root canal preparation” AND “tooth, deciduous”)	
	TITLE-ABS-KEY (“root canal therapy” OR “root canal preparation” AND “anti-bacterial agents” AND “tooth, deciduous”)	
	TITLE-ABS-KEY (“metronidazole” OR “ciprofloxacin” OR “minocycline” OR “tinidazole” “tetracycline” OR “agents anti-bacterial” OR “agentsantibacterial” OR “agentsantimycobacterial” OR “antibiotic” OR “antibiotic paste” AND “deciduous teeth”)	
	TITLE-ABS-KEY (“root canal preparation” OR “canal preparation root” OR “root canal therapy” OR “canal therapies root” OR “root canal procedures” OR “root canal treatment” OR “lstr” OR “non-instrumentation endodontic” AND “deciduous teeth”)	

**Table 2:** Characteristics of the included studies.

Study	Study design	Subjects (no. of children/age in years/no. of teeth)	Primary teeth selected	Results				Conclusions
				Antibiotics mixture	Follow-up (months)	Clinical success *N* (%)	Radiograph success *N* (%)	
Pinky et al. [13] India/2011	RCT	28 children/4–10 years/40 primary teeth	Primary molars	Ciprofloxacin-minocycline-metronidazole	3 6 12	20/20 (100) 20/20 (100) 18/20 (90)	20/20 (100) 20/20 (100) 18/20 (90)	Good clinical and radiographic success in both groups. No statistically significant difference between the two groups (*P* > 0.05).
				Ciprofloxacin-minocycline-ornidazole		20/20 (100) 20/20 (100) 20/20 (100)	20/20 (100) 20/20 (100) 20/20 (100)	

Nanda et al. [14] India/2014	RCT	38 children/4–10 years/40 primary teeth	Primary molars	Ciprofloxacin-minocycline-metronidazole	3 6 12	20/20 (100) 20/20 (100) 20/20 (100)	20/20 (100) 20/20 (100) 17/20 (85)	100% clinical success in both groups. Radiological changes of two groups. Statistically similar observations in both groups (*X*^*2*^ = 1.35, *P*=0.509). Radiological success rate was statistically similar in both groups (*P*=0.613).
				Ciprofloxacin-minocycline-ornidazole		20/20 (100) 20/20 (100) 20/20 (100)	20/20 (100) 20/20 (100) 19/20 (95)	

Singh et al. [16] India/2017	RCT	38 children/5–10 years/80 primary teeth	Primary teeth	Ciprofloxacin-metronidazole-minocycline	3 6 12	40/40 (100) 40/40 (100) 36/40 (90)	Nm Nm 40/40 (100) 36/40 (90)	No statistically significant difference between both groups. The ciprofloxacin-minocycline-ornidazole group showed better results clinically and radiographically compared with the 3 Mix group.
				Ciprofloxacin-minocycline-ornidazole		40/40 (100) 40/40 (100) 40/40 (100)	Nm Nm 40/40 (100) 40/40 (100)	

Jaya et al. [15] India/2012	RCT	25 children/6–9 years/30 primary teeth	Primary molars	Ciprofloxacin-metronidazole-minocycline	6 12 24	15/15 (100) 15/15 (100) 15/15 (100)	9/15 (60) 9/15 (60) 9/15 (60)	Good clinical and radiographic success in both groups. No statistically significant difference between the two groups (*P* > 0.05).
				Ciprofloxacin-tinidazole-minocycline		15/15 (100) 15/15 (100) 15/15 (100)	8/15 (53.3) 8/15 (53.3) 8/15 (53.3)	

Raslan et al. [17] Syria/2017	RCT	22 children/Nm/42 primary teeth	Mandibular primary molars	Ciprofloxacin-metronidazole-minocycline	1 3 6 12	21/21 (100) 19/21 (90.4) 19/19 (100) 19/19 (100)	21/21 (100) 19/21 (90.4) 17/19 (89.4) 11/19 (89.4)	No statistically significant differences Between the two groups regarding the appearance of radiolucency and the radiographic success rates after 6 and 12 months of treatment (*P* > 0.05). Regarding the association between root resorption degree and the clinical and radiographic success of the treatment within each group individually during all the follow-up periods (*P* > 0.05).
				Ciprofloxacin-metronidazole-clindamycin		21/21 (100) 20/21 (95.2) 20/20 (100) 20/20 (100)	21/21 (100) 20/21 (95.2) 16/20 (80) 11/20 (55)	

RCT: randomized controlled study; Nm: not mentioned.

**Table 3:** Summary of the results drawn from selected studies at the 12-month follow-up.

Study	ATB mixture	Vehicle	Ratio	Irrigants	Restoration	Final follow-up (months)	Clinical outcomes					Exfoliation	Radiographical outcomes		
							Pain	Swelling/fistula	Abscess	Pain in percussion	Mobility		Bone regeneration	Increased radiolucency	No changes
Pinky et al. [13] India/2011	Ciprofloxacin-metronidazole-minocycline	Propylene glycol	1 : 3 : 3	Saline	Temporary dressing with zinc oxide eugenol/stainless steel crowns (after 30 days)	12	2 (10%)	—	—	—	—	—	11 (55%)	2 (10%)	7 (35%)
	Ciprofloxacin-minocycline-ornidazole		1 : 3 : 3				—	—	—	—	—	—	12 (60%)	—	8 (40%)
Nanda et al. [14] India/2014	Ciprofloxacin-metronidazole-minocycline	Propylene glycol	1 : 3 : 3	Saline	Glass ionomer cement/stainless steel crowns (at the same visit)	12	—	—	—	—	—	—	6 (30%)	3 (15%)	7 (35%)
	Ciprofloxacin-minocycline-ornidazole		1 : 3 : 3				—	—	—	—	—		7 (35%)	4 (20%)	1 (5%)
Singh et al. [16] India/2017	Ciprofloxacin-metronidazole-minocycline	Propylene glycol	1 : 3 : 3	Saline	Temporary dressing (zinc oxide eugenol/glass ionomer cement (after 15 days)/stainless steel crowns (after 30 days))	12	2 (5%)	—	—	—	2 (5%)	—	22 (55%)	4 (10%)	14 (35%)
	Ciprofloxacin-minocycline-ornidazole		1 : 3 : 3				—	—	—	—	—		24 (60%)	—	16 (14%)
Jaya et al. [15] India/2012	Ciprofloxacin-metronidazole-minocycline	Propylene glycol/macrogol	1 : 3 : 3	Saline	Glass ionomer cement/stainless steel crowns (at the same visit)	24	—	—	—	—	8 (62%).	—	Nm	9 (69%).	Nm
	Ciprofloxacin-tinidazole-minocycline		1 : 3 : 3				—	—	—	—	7 (50%)		Nm	8 (57%)	Nm
Raslan et al. [17] Syria/2017	Ciprofloxacin-metronidazole-minocycline	Propylene glycol	1 : 1 : 1	Phosphoric acid/sterile water	Glass ionomer cement/stainless steel crowns (at the same visit)	12	—	1 (3, 6%)	2 (9, 52%)	—	—	3 (16.67%)	11 (61.11%)	1 (5.56%)	3 (16.67)
	Ciprofloxacin-metronidazole-clindamycin		1 : 1 : 1				—	—	1 (3, 52%)	—	—	2 (10%)	11 (55%)	4 (20%)	3 (15%)

The symbol “—” indicates absent; Nm: not mentioned.

## Data Availability

All data generated and analysed are included within this published article.

## Additional Points

This paper is a systematic review of the literature, and no experiments involving humans or animals were performed by any of the authors.
